# Triage and dysphagia: Are hospitals in the South African public health sector ready?

**DOI:** 10.4102/sajcd.v69i1.852

**Published:** 2022-06-30

**Authors:** Kelly-Ann Kater

**Affiliations:** 1 Department of Speech Pathology and Audiology, Faculty of Humanities, University of the Witwatersrand, Johannesburg, South Africa

**Keywords:** dysphagia, emergency department, triage, speech-language therapy, evidence-based practice, dysphagia triage

## Abstract

Dysphagia screening is unequivocally beneficial for individuals who may be at risk of swallowing impairment. Benefits range from capitalising on early intervention, facilitating hydration and nutrition, reduced financial costs for the patient and prevention of dysphagia-related complications. Why then is there a need for triage? Inefficiencies and often non-existence of screening and referral processes require one to consider if triage may be a more viable option in the public healthcare context. Dysphagia triage could potentially prioritise emergency swallowing care and identify patients who need immediate swallowing attention because of the nature or severity of dysphagia. The use of a dysphagia triage checklist could have implications for patient health outcomes in terms of the safety of oral diets, development of aspiration pneumonia, malnutrition, administration of oral medication and overall patient prognosis.

## Introduction

Dysphagia is a common cause of aspiration and subsequently a common cause of morbidity and mortality (White, O’ Rourke, Ong, Cordato, & Chan, 2008). Early detection of dysphagia is essential to reduce length of hospitalisation, degree of morbidity, hospital costs and the risk of pneumonia (Ostrofsky & Seedat, 2016). The emergency department (ED), being the first entry point for any patient into the hospital, could be the most opportune time and space to triage a patient for dysphagia. Triaging patients enables identification of patients at risk of dysphagia. Early detection of dysphagia improves health outcomes.

Given the South African hospital context, the limited number of speech-language therapists (SLTs) practicing in the public sector, the working hours of SLTs in hospital settings and the high patient-to-therapist ratio, triaging for dysphagia in the ED could facilitate identification of patients at risk of dysphagia, which in turn may enable early and appropriate referral to a SLT.

## The South African public health sector

The South African public health sector caters to approximately 84% of the total population, the majority of whom are poor and uneducated (Naidoo, 2012). ‘The inequalities and poor quality in the health system are aggravated by a skewed distribution of key health professionals between the public and private sectors’ (Department of Health [DOH], 2017).

[*T*]he shortage of key health professionals is being experienced in a time of the growth of the population dependent on public healthcare services, and the increasing burden of disease among the population. (p. 23)

This has placed extraordinary strain on public sector health services and on the staff who work in public sector facilities. This, in turn, has contributed to the poor health outcomes of South Africans, particularly for the lowest income populations and households.

In South Africa, there are insufficient SLTs to manage the patient demand (Pillay, Tiwari, Kathard, & Chikte, 2020), with only a small percentage actually providing comprehensive dysphagia services (Andrews & Pillay, 2017). Dysphagia services have evolved in response to the increasing evidence base for the role of the multidisciplinary team (MDT) members in screening for dysphagia and the role of SLT in the assessment, diagnosis and management of dysphagia (Brady & Roe, 2020). However, dysphagia care pathway designs and provision are hugely driven by service capacity or national guidance (Brady & Roe, 2020). As the National Health Insurance (NHI) is still within the planning phase, it may prove to be beneficial for dysphagia triage to be included in the care pathway for dysphagia.

## National Health Insurance

[*The*] NHI is a health care financing system that is designed to pool funds to actively purchase and provide access to quality, affordable personal healthcare services for all South Africans based on their health needs, irrespective of their socioeconomic status. (DOH, 2017, p. 13)

The NHI aims to cover services that are delivered on a people-centred integrated healthcare system (DOH, 2017). This will require a more responsive and accountable health system. The health system would need to consider sociocultural and socioeconomic factors. In addition, this people-centred integrated healthcare system platform aims to improve user satisfaction and in turn should lead to a better quality of life and improved health outcomes across all socioeconomic backgrounds (DOH, 2017).

The features of the NHI include efficiency and effectiveness. This means that healthcare resources should be allocated and utilised in a manner that optimises value for money by using the resources to maximum advantage and by maximising the welfare of the community by achieving the right mixture of healthcare programmes for the entire population (DOH, 2017). The NHI further aims to ensure that the health system meets acceptable standards of quality and achieves positive health outcomes (DOH, 2017).

On paper, the concept of dysphagia triage fits into the NHI framework, however, it appears as if the South African health context has a significant way to go in terms of staff numbers and access to resources before these systems may be effective. Furthermore, international research is moving towards the notion of models of care and ‘the ideal patient journey’ (New South Wales [NSW] Health, 2012). This concept is based on the following:

1. Early assessment and streaming to an appropriate model of care
2. A team approach to patient care
3. Ensuring tasks are performed by the medical practitioner who can most efficiently perform the task (where ‘efficiency’ balances quality, cost and minimising duplication of work)
4. Coordinated patient care
5. Strong monitoring and evaluation measures
6. Adherence to the principles of models of care (NSW Health, 2012).

Looking at these principles of the ideal patient journey, early identification of dysphagia in at-risk patients would fit well into this model (Barnard, 2011). Whilst in theory and based on the literature, dysphagia triage is of value to the acute health system, it is unlikely at this stage that South African EDs would be able to cope with the additional demand of the dysphagia triage checklist, as the focus of any ED is to stabilise medically unstable patients, with the risk of dysphagia and aspiration unlikely to be a priority at that time.

## The speech-language therapist in South Africa

Screening, assessment, diagnosis and management of dysphagia typically falls under the scope of practice of the SLT because of their training in the anatomy, neuroanatomy and physiology of deglutition (Cichero, Heaton, & Bassett, 2009; Logemann, 1998). The aims and objectives of the SLTs’ interventions for dysphagia depend on the type and nature of the dysphagia, the underlying cause and the needs and preferences of the individual (Cichero et al., 2009). However, because of the high patient-to-therapist ratio (Pillay et al., 2020), limited resources and late patient referrals, screening may not be regularly conducted in South African hospitals within the public sector. The dysphagia guideline set out by the South African Speech Language Hearing Association (SASLHA), advocates for screening procedures to be conducted by other MDT members, which includes but is not limited to doctors and nurses (SASLHA, 2011). Currently, it is likely that screening may be carried out by SLTs on an ad hoc basis, depending on time, staffing and the availability of students in training from universities who may be of assistance. Despite there being several South African and global dysphagia screeners, no standardised national screening guideline for dysphagia exists, suggesting that each context or hospital within the public healthcare sector may be operating in line with their institutions’ human resources, patient numbers and logistical availability. Like the universal newborn hearing screening guidelines which exist for audiologists, similar studies pertaining to dysphagia have not been undertaken or published. This risk-based hearing screening programme has been found to be practical and feasible for South Africa (Kanji & Khoza-Shangase, 2016). However, the inefficiencies and often non-existence of dysphagia screening and referral processes require one to consider if triage may be a more viable option in the public healthcare context. Considering the safety of swallowing, managing aspiration and preventing complications are of utmost importance (Logemann, 1998).

A policy developed by the Royal College of Speech and Language Therapists (RCSLT) in the United Kingdom recommends that there should be at least one full-time SLT per 10 beds in every stroke unit (RCSLT, 2007). Furthermore, the policy suggests that if the ratio of SLTs per 10 patients falls below this then it would impede timely assessments and follow-up management (RCSLT, 2007). However, SLTs in South Africa comprise a small percentage of practicing healthcare professionals and often find themselves trying to service large caseloads in a country that has limited resources (Blackwell & Littlejohns, 2010; Pillay et al., 2020). In addition, SLTs are most commonly available during regular working hours on weekdays. Thus, nurses and doctors play a pivotal role in patient care and communication between other professionals as they provide 24-h care. South African Speech Language Hearing Association recommends that the management of dysphagia requires MDT involvement with regard to screening; however, assessment is within the scope of practice of the SLT (SASLHA, 2011).

## Dysphagia triage

Dysphagia triage could potentially prioritise emergency swallowing care and identify patients who need immediate attention because of the nature or severity of dysphagia. In order to understand the concept of triaging it is important to differentiate between triaging and screening. Triage refers to identifying the possibility of the presence or absence of a dysphagia and does not involve food trials, whereas screening examines (1) the likelihood of dysphagia, (2) the requirement for further swallowing assessment, (3) the safety of patient oral intake and (4) the requirement for alternative nutritional support. It is proposed that the dysphagia triage checklist takes place at the earliest point in the patient’s presentation to the hospital (i.e. the ED). Triaging would require minimal training, could be administered in less than 2 min and does not use food trials. It can be administered by any medical professional and can be used on a variety of patient populations and is not limited to a specific diagnosis (e.g. stroke). A fail result for any of the test items indicates that the patient is at risk of dysphagia and therefore a screening is indicated.

The literature advocates for dysphagia triage; however, context and environmental circumstances need to be considered. The knowledge that early interventions may prevent dysphagia complications leads to the recognition that it would be worthwhile to perform dysphagia triaging in the ED (Barnard, 2011). Personnel in the ED have the potential to affect the patient care in a positive way, advocate for early recognition of the signs and symptoms of dysphagia and potentially guide the management of this disorder (Barnard, 2011). However, the implementation of a dysphagia triage protocol goes beyond recommendations from international literature and is hugely dependent on the context, particularly in South Africa.

## Challenges with early dysphagia identification

There are many challenges surrounding effective identification of patients who present with dysphagia in developing contexts. These challenges include timing of identification of a swallowing difficulty and the timing of the assessment (Ostrofsky & Seedat, 2016; Seedat, 2014). Thus, clinicians are faced with using current research and evidence-based practice to make decisions for patients and to demonstrate that this research improves clinical outcomes (Jones, Roop, Pohar, Albrecht, & Scott, 2015). ‘Knowledge translation is an active process that facilitates the introduction of new evidence into practice and may identify optimum strategies to close the gap between research and clinical practice’ (Jones et al., 2015, p. 2).

The dynamics of knowledge flow are dependent on many factors, some of which include the nature of the research and the targeted level of implementation, whether it be implemented at a clinical or organisational level (Jones et al., 2015). Changing clinical behaviour (in this case, the implementation of the dysphagia triage checklist) is dependent not only on individual factors but also factors such as hospital policy, funding and organisational or management influence and staff workload (Jones et al., 2015). Thus, the implementation of a tool such as the dysphagia triage checklist requires a bottom-up method of implementation, as it requires the buy-in from both management and staff on the ground level.

## Conclusion

Early identification of dysphagia has implications for patient management and outcomes. As a result of the poor prognostic outcome of patients with dysphagia, the need to identify reasonable means of early identification is imperative. Inefficiencies and often non-existence of screening and referral processes require one to consider if triage may be a more viable option in the public healthcare context. Dysphagia triage could potentially prioritise emergency swallowing care and identify patients who need immediate swallowing attention because of the nature or severity of dysphagia and would help to combat inequities, such as the limited number of SLTs practicing in the public sector, the availability of SLTs outside of working hours and the high patient-to-therapist ratios. However, given the current status of the healthcare system in South Africa, that is, poorly resourced, over-crowded, understaffed and underfunded (DOH, 2017), it is uncertain if a dysphagia triage protocol could be accommodated within the current functioning of the accident and emergency unit.
